# The Effect of Diabetes Self-Management Education on Glycemic Control in Minority Patients With Diabetes Mellitus

**DOI:** 10.7759/cureus.16888

**Published:** 2021-08-04

**Authors:** Xiu Ying Au, Sneha Kola, Vinuta Mohan

**Affiliations:** 1 Internal Medicine, Saint Francis Medical Center, Trenton, USA

**Keywords:** retrospective study, glycemic control, hemoglobin a1c, new jersey, diabetes mellitus, diabetes self-management education, hispanic, non-hispanic black

## Abstract

Background

Diabetes self-management education (DSME) plays a vital role in diabetes control yet is highly underutilized, especially in the minority population. The efficacy of DSME on glycemic control among the Hispanic and non-Hispanic black population is not as well established as it is compared to the non-Hispanic White population.

Methodology

In this retrospective cohort study, patients who participated in both group and one-to-one DSME classes at Saint Francis Medical Center, Trenton, New Jersey, from 2017 to 2019 were identified. Mean hemoglobin A1c levels before DSME and after DSME were compared using paired t-test.

Results

A total of 344 patients were included in the study. Out of 344 patients, 200 (58%) patients were Hispanic and 97 (28%) were non-Hispanic black, 42 (12%) were Caucasian, and five (2%) were from other races. The mean hemoglobin A1c was reduced by an average of 1.08% among patients who participated in group DSME (DSME done in group settings) and by an average of 1.95% among patients who participated in one-to-one DSME (DSME done in individualized settings).

Conclusion

DSME is effective in reducing hemoglobin A1c levels in Hispanic and non-Hispanic black majority patients. One-to-one DSME is more effective than group DSME in reducing hemoglobin A1c in this patient population.

## Introduction

Uncontrolled diabetes is related to multiple macrovascular and microvascular complications, which leads to a significant amount of healthcare cost. The estimated total economic cost of diagnosed diabetes in 2017 was $327 billion [1]. Achievement of better glycemic control is essential in reducing the healthcare cost related to diabetes. Lack of diabetes self-management education (DSME) is identified as one of the main reasons for uncontrolled diabetes. DSME is the ongoing process of facilitating the knowledge, skill, and ability necessary for diabetes self-care in active collaboration with the health care team. Unfortunately, studies have found that DSME has a low participation rate; only 55.3% of all people with diabetes have ever attended a DSME program in 2016 [2].

According to the 2020 Center for Disease Control and Prevention (CDC) National Diabetes Statistic Report, 34.2 million of the population in the United States are diagnosed with diabetes, up to 11.9 million (34.8%) are non-Hispanic blacks, Hispanics, and non-Hispanic Asian [3]. However, the efficacy of DSME in minorities has not been studied as much as in non-Hispanic white patients. Only a small number of studies investigated the efficacy of DSME among Hispanic and non-Hispanic black patients [4-7]. Among those studies, a randomized-controlled trial conducted by Spencer et al. showed the best results, during which the mean hemoglobin A1c of African American and Latino adults with type 2 diabetes was reduced by 0.8% after six months of DSME [5]. 

There was also a lack of data that compares the efficacy of individual DSME versus group DSME among the minority ethnicities. Llorca et al. performed a randomized-controlled trial, which revealed no difference in effectiveness between the group and individual DSME among Latino patients [8]. Therefore, the objective of this retrospective cohort study was to determine the efficacy of DSME on glycemic control in a predominantly non-Hispanic black and Hispanic patient population.

## Materials and methods

This is a retrospective cohort study conducted at Saint Francis Medical Center in Trenton, New Jersey. Patients with diabetes who ever participated in DSME from 2017 to 2019 were identified. There were two exclusion criteria for this study. First, patients who had baseline hemoglobin A1c of less than 5.7% were excluded. Therefore, patients who did not fulfill diagnostic criteria of diabetes or prediabetes based on hemoglobin A1c were excluded from the study. Second, patients who did not have a follow-up hemoglobin A1c level performed between 1.5 and 24 months after DSME completion were excluded. The paper health records were obtained from archives in the Department of Health Information Management of Saint Francis Medical Center, while electronic health records were obtained from the electronic medical record systems used in Saint Francis Medical Center, Soarian (North Kansas City, MO: Cerner Corp.), and NextGen (Atlanta, GA: NextGen Healthcare, Inc.). Data collection sheets were created, which include the following information: age, gender, ethnicity, type of DSME participated (i.e., group DSME versus one-to-one DSME), and hemoglobin A1c before and after DSME. Spreadsheets without patients’ identifiers were uploaded for data analysis. Data analysis was performed using statistical analysis software, SAS (Cary, NC: SAS Institute), and pvalue.io (Paris, France: Medistica, EURL {LLC}) [9]. Hemoglobin A1c before and after DSME was compared using paired t-test. The p-value of the study was <0.05, which indicates statistical significance. Demographic characteristics such as age group, gender, and ethnicity of research subjects were also summarized using a table. 

## Results

**Figure 1:**
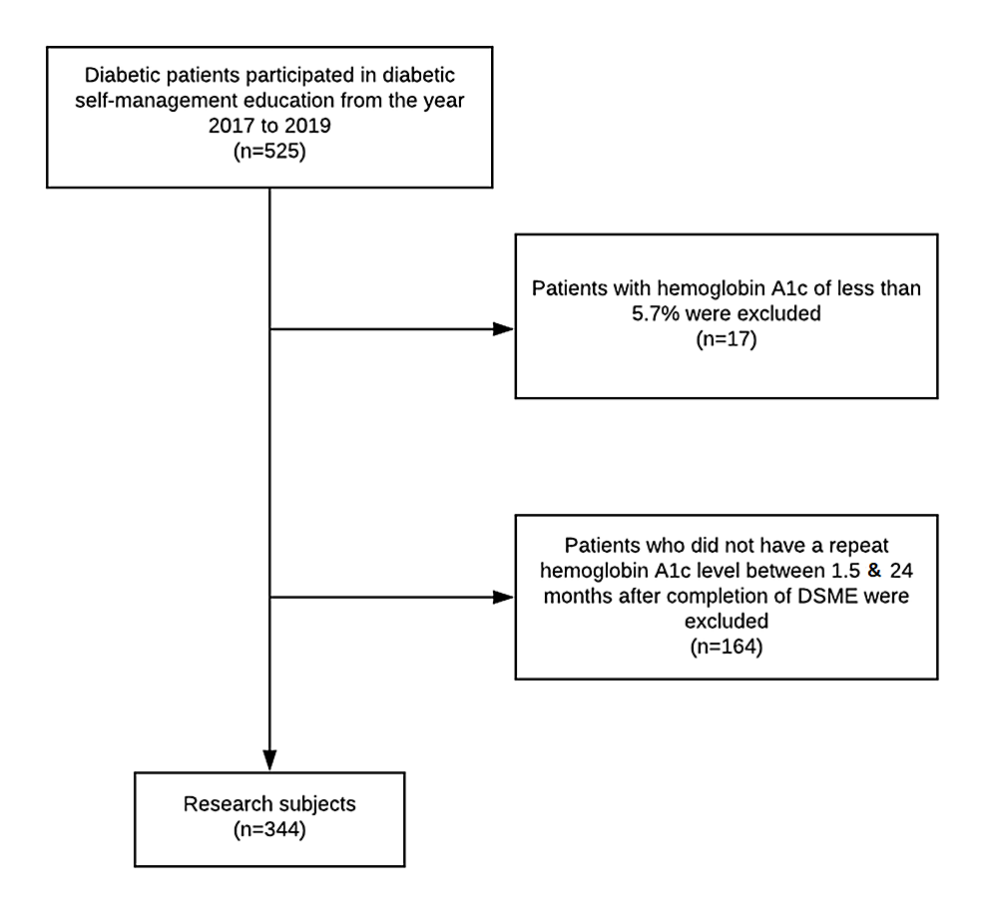
Selection of study sample

A total of 525 patients with diabetes who participated in DSME between January 2017 and December 2019 were identified. Among 525 patients, 17 patients whose baseline hemoglobin A1c values were less than 5.7% were excluded from the study, and 164 patients who did not have a repeat hemoglobin A1c value between 1.5 and 24 months after completion of DSME were excluded (Figure 1). 

Patients were allowed to choose between group DSME or one-to-one DSME. There were 279 patients who enrolled into group DSME, while there were 65 patients who decided to participate in one-to-one DSME. Each class of group DSME has an average of 10 patients to ensure that the patients-to-educator ratio is no more than 20:1. 

Table 1 demonstrates the demographic characteristics of the study sample. Of the 344 patients who were included in the study, 158 (46%) were female. The age group with the largest number of patients is 50-59 years, with 140 (41%) patients. Fifty-eight percent (200) of the patients were Hispanic, and 28% were non-Hispanic black. 

**Table 1:** Demographic characteristics of study sample

Characteristics	Number of patients (%)
Age	
<40	23 (7%)
40-49	78 (23%)
50-59	140 (41%)
60-69	81 (23%)
70-79	19 (6%)
≥80	3 (1%)
Gender	
Female	158 (46%)
Ethnic group	
Caucasian	42 (12%)
Non-Hispanic black	97 (28%)
Hispanic	200 (58%)
Other	5 (2%)

**Figure 2:**
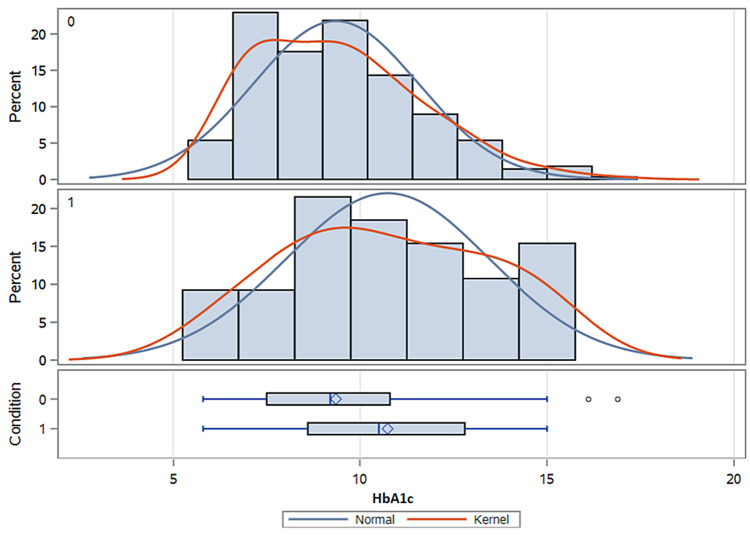
Distribution of baseline hemoglobin A1c of group DSME (top Bell curve) versus one-to-one DSME (bottom Bell curve) DSME: diabetes self-management education; HbA1c: glycosylated hemoglobin

Mean baseline hemoglobin A1cs was 10.7% in participants of one-to-one DSME, which was 1.3% higher than participants of group DSME (Table 2, Figure 2).

**Table 2:** Characteristics of baseline hemoglobin A1c in group DSME versus one-to-one DSME DSME: diabetes self-management education

Type of DSME	Mean (SD)	Median (Q25-75)	Min	Max	Number
Group DSME	9.35 (2.20)	9.20 (7.50; 10.8)	5.80	16.9	279
One-to-one DSME	10.7 (2.72)	10.5 (8.60; 12.8)	5.80	15.0	65

**Figure 3:**
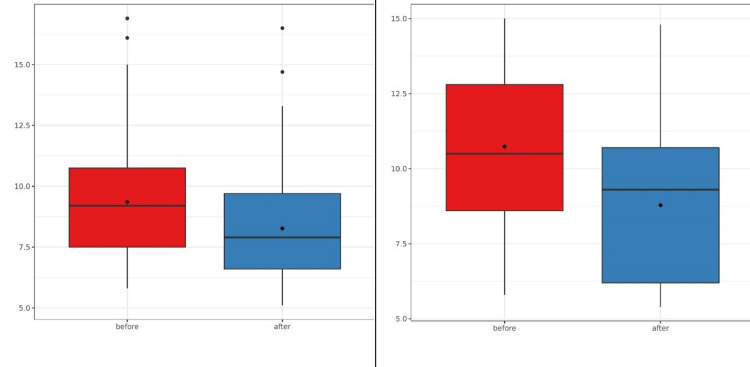
Comparison of hemoglobin A1c before and after group DSME (left) and one-to-one DSME (right). The p-values of both before-after analyses are <0.001. DSME: diabetes self-management education

Mean hemoglobin A1c was reduced by 1.08% (95% CI, 1.36 to 0.81; p<0.01) among participants of group DSME and by 1.95% (95% CI, 1.17 to 2.73; p<0.001) among participants of one-to-one DSME (Table 3, Figure 3). 

**Table 3:** Comparison of hemoglobin A1c before and after group and individual DSME DSME: diabetes self-management education

Type of DSME	Before A1c, mean	After A1c, mean	Δ mean A1c (95% confidence interval)	Number	p-Value
Group DSME	9.35	8.27	-1.08 (-1.36-0.81)	279	<0.001
One-to-one DSME	10.73	8.78	-1.95 (-1.17-2.73)	65	<0.001

## Discussion

Ethnic minorities are found to have more insufficient glycemic control [10]. A meta-analysis has shown that in comparison to non-Hispanic patients, Hispanic patients have approximately 0.5% higher hemoglobin A1c [11]. Cross-sectional studies revealed that African American patients have more inadequate diabetes control than their white counterparts [11]. Lack of physical activity, poorer eating habits, and lower rates of self-monitoring of blood glucose among ethnic minorities were the contributors to ethnic disparities in glycemic control [12]. Due to this fact, DSME, which facilitates the knowledge and behaviors of diabetes self-care, becomes an important component in achieving glycemic control among ethnic minorities. Our retrospective cohort study observed a reduction in mean hemoglobin A1c level by 1.08% after completion of group DSME in a Hispanic and non-Hispanic black majority population. This result is comparable to the previous meta-analysis, which has proved the efficacy of DSME in improving hemoglobin A1c levels in a predominantly English-speaking population [13]. The result of our study is more robust than the prior study conducted by Spencer et al., which showed a reduction in hemoglobin A1c of 0.8% among African American and Latino populations [5]. When comparing group DSME versus one-to-one DSME, our study proved that one-to-one DSME resulted in at least an additional 0.87% reduction in mean hemoglobin A1c level. Our result is opposite to the previous research performed by Rickheim et al. demonstrated that hemoglobin A1c improvement in participants of group DSME is marginally greater than those who participated in individual DSME [14]. The reason behind this contradiction might be that the patients who participated in one-to-one DSME received personalized education and advanced education material, while group DSME participants did not. A 1% reduction in hemoglobin A1c would benefit ethnic minorities with diabetes both financially and clinically. One percent reduction is associated with reducing the risk of microvascular complications by 37%, myocardial infarction by 14%, and subsequently diabetes-related death by 21% [15]. Reduction in complications of diabetes would also lead to reductions in healthcare costs. Based on a claims analysis performed in 2013, a 1% reduction in hemoglobin A1c could lead to $1169 decrease in the cost of care for each patient [16]. This reduction in patient-level healthcare cost is significant for ethnic minorities, with a higher proportion of patients having a lower socioeconomic status. A cross-sectional study has revealed that in New Jersey, up to 65.2% of Hispanic patients and 72.9% of patients of other non-Hispanic ethnic minorities have never participated in DSME [17]. Strategies employed in our medical center to promote the participation of DSME in our patient population have led to our robust results. Our DSME classes were available at different times of the day, including after-work hours. This strategy encouraged DSME participation in those patients who had missed DSME classes because of work or family-related causes. Also, DSME classes were available in English and Spanish, which overcame the language barrier in diabetes education among minorities. In addition, our DSME educators also provided classes that were culturally adapted as staple foods vary between the Hispanic and non-Hispanic populations, which facilitated the practice of healthier eating habits among the participants with different cultural backgrounds.

Furthermore, our DSME classes have an average of only 10 participants per class. The smaller class size allowed more interactions between DSME educators and each participant. Finally, incentives such as gift bags and food vouchers were provided to DSME attendees to encourage participation in DSME classes further. The sample size of the study limits the generalizability of the results. It was beyond the scope of this study to demonstrate the relationship between DSME participation hours and glycemic control. Follow-up hemoglobin A1c values were obtained after six weeks to 24 months, and the follow-up intervals varied between patients. Variability in the duration of follow-up limited the study to determine the immediate effect and the long-term effect of DSME on glycemic control. Further research is needed to establish the structure of DSME that is effective for glycemic control among ethnic minorities. Ways to maintain the long-term effect of DSME on glycemic control must also be investigated. 

## Conclusions

Our study successfully proves that DSME is effective in improving glycemic control in Hispanic and non-Hispanic black patients. Although DSME is recommended for all patients with diabetes as per the most recent American Diabetes Association guidelines, DSME participation rates are especially low. All patients with diabetes, especially those from ethnic minorities, should be referred by their primary care physician for DSME annually for learning or reinforcement of DSME. Multiple tactics to improve their diabetes control are required to promote the broader utilization of DSME, especially among the Hispanic population. 
